# Effect of β-Eudesmol on NQO1 suppression-enhanced sensitivity of cholangiocarcinoma cells to chemotherapeutic agents

**DOI:** 10.1186/s40360-018-0223-4

**Published:** 2018-06-19

**Authors:** Pimradasiri Srijiwangsa, Saranyoo Ponnikorn, Kesara Na-Bangchang

**Affiliations:** 1 Chulabhorn International College of Medicine, Thammasat University, (Rangsit Campus), Pathum Thani, 12121 Thailand; 2 Center of Excellence in Pharmacology and Molecular Biology of Malaria and Cholangiocarcinoma, Chulabhorn International College of Medicine, Thammasat University, Pathum Thani, Thailand

**Keywords:** Cholangiocarcinoma, NAD(P)H-quinone oxidoreductase 1, β-Eudesmol, 5-Fluorouracil, Doxorubicin, Apoptosis, Migration

## Abstract

**Background:**

Cholangiocarcinoma (CCA), an epithelial malignancy of the biliary tree, is one of the aggressive cancers with poor prognosis and unsatisfactory response to chemotherapy with acquired resistance. NAD(P)H-quinone oxidoreductase 1 (NQO1), an antioxidant/detoxifying enzyme, plays important roles in chemo-resistance and proliferation in several cancer cells. The study aimed to investigate the inhibitory effect of β-eudesmol on NQO1 enhanced chemotherapeutic effects of 5-fluorouracil (5-FU) and doxorubicin (DOX) in the high NQO1-expressing human CCA cell line, NQO1-KKU-100. In addition, the molecular events associated with the inhibition of the cell proliferation, cell migration, and induction of apoptosis were investigated.

**Methods:**

Human CCA KKU-100 cells were exposed to β-eudesmol at various concentrations. NQO1 enzyme activity and protein expression were measured by enzymatic assay and Western blot analysis, respectively. Sulforhodamine B (SRB) assay and wound healing assay were performed to detect the inhibitory effect of β-eudesmol on cell proliferation, cell migration, and sensitivity to 5-FU and DOX. Apoptotic induction was detected by flow cytometry with annexin V/PI and DAPI nuclear staining. Caspase 3/7 activation was determined by fluorescence microscopy. The mechanism of enhanced chemo-sensitivity was evaluated by Western blot analysis.

**Results:**

β-Eudesmol significantly suppressed NQO1 enzyme activity (both in KKU-100 cells and cell lysates) and protein expression in KKU-100 cells in a concentration-dependent manner. β-Eudesmol exhibited potent cytotoxicity on KKU-100 cells with mean ± SD IC_50_ values of 47.62 ± 9.54 and 37.46 ± 12.58 μM at 24 and 48 h, respectively. In addition, it also potentiated the cytotoxic activities and inhibitory activities of 5-FU and DOX on cell migration through induction of cell apoptosis and activation of caspase 3/7. Western blot analysis suggested that β-eudesmol enhanced chemosensitivity was associated with the suppression of NQO1 protein and activation of Bax/Bcl-2 protein expression ratio in CCA cells.

**Conclusions:**

β-Eudesmol may serve as a potential anti-CCA candidate particularly when used in combination with conventional chemotherapeutics. The mechanisms involved may be mediated via NQO1 suppression-related apoptosis pathway.

## Background

Cholangiocarcinoma (CCA) is an extremely aggressive malignant tumor of the bile duct that becomes one of the major health problems worldwide. It is originating from the epithelial cells of the extrahepatic or intrahepatic bile ducts [1]. The northeastern region of Thailand including countries around Mekong basin are documented as areas with highest CCA incidence in the world [2, 3]. Epidemiological and experimental studies provide evidence that chronic inflammation and cell injury as a consequence of infection with the liver flukes *Opisthorchis viverrini* or *Chlonorchis sinensis* is the main risk factor of CCA [4]. The diagnosis of CCA is challenging because most patients are present with progressive and advanced stages resulting in disease poor prognosis [5]. Currently, management of CCA remains a challenge because the only occasional therapy is surgical resection. Chemoresistance is the major obstacle in the treatment of CCA particularly in unresectable tumors [6]. Multiple mechanisms involved in resistance of CCA to chemotherapeutic agents have been proposed. These include alteration of drug metabolizing enzymes, efflux transporters, cytoprotective enzymes, or derangement of intracellular signaling system [7, 8]. Novel effective therapy to overcome the chemoresistance of CCA is urgently needed.

NAD(P)H-quinone oxidoreductase 1 (NQO1; EC 1.6.5.2) is mainly a cytosolic phase II detoxification enzyme that reduces quinones to hydroquinones and thus bypassing the toxic semiquinone intermediates. The resultant hydroquinones undergo further conjugation and excretion [9]. NQO1 is ubiquitously expressed at low basal levels in all types of normal human tissues except liver through Nrf2 dependent pathway and proteasome degradation [10, 11]. The enzyme is induced along with a battery of defensive enzymes in response to cellular stress to prevent carcinogenesis process in body tissues through its free radical scavenging activity [9, 12, 13]. Conversely, the expression of NQO1 has been found to be increased in cancers of lung [14], pancreas [15], breast [16], thyroid [17], stomach [18], and bile duct (CCA) [19]. It is hypothesized that high level of NQO1 expression promotes carcinogenesis and cancer progression while also making cells more resistant to anticancer drugs particularly oxidative stress inducers. The critical role of NQO1 as a promising target for cancer chemotherapy has been demonstrated in various studies. Inhibition of NQO1 activity by dicoumarol, the pharmacological NQO1 inhibitor, was shown to suppress urogenital cancer cell growth and potentiate cytotoxicity of doxorubicin and cisplatin [20, 21]. In CCA, dicoumarol was shown to potentiate gemcitabine-induced cytotoxicity in the high NQO1-expressing CCA [22]. Furthermore, knocking down of NQO1 gene expression by small interfering RNA (siRNA) in the high NQO1-expressing CCA cells was shown to enhance the cytotoxic effect of 5-fluorouracil, doxorubicin, and gemcitabine [23]. Searching for specific NQO1 inhibitors would therefore be one of the promising approaches for discovery and development of new chemotherapeutics for CCA. A large number of moderate to potent NQO1 inhibitors from natural and synthetic sources have been reported including flavonoids, coumarins, curcumin, and ES936, of which the most demonstrative inhibitors are dicoumarol and ES936 [24–26]. Dicoumarol acts by completing with NAD(P)H and thereby preventing the reduction of FAD in cells. The compound is commonly used to investigate the inhibitory effect on NQO1 activity and its consequences in several cell types. Based on X-crystallography, the conformational change of the amino acid residues Tyr128 and Phe232 located in the catalytic pocket of NQO1 have been demonstrated upon binding of dicoumarol [27]. Unlike dicoumarol, flavonoids have been identified as strong NQO1 inhibitors through competitive inhibition of NAD(P)H [27]. Preliminary molecular dynamic studies suggested that flavonoids bind to different active site of NQO1 from that of dicoumarol; the 7-hydroxyl moiety of the flavonoids interacts with His161 residue in the active site [28]. The inhibition of NQO1 by flavonoids is pointing at a mechanism contradicting the proven beneficial properties of these phytochemicals. Novel NQO1 inhibitors are being identified by a structure-based or mechanism-based approach using reference NQO1 inhibitors.

β-Eudesmol is a one of the major constituents of the rhizome of *Atractylodes lancea* (Thunb) DC. (AL: Khod-Kha-Mao or Cang Zhu) of the Compositae family. It is the common medical plant used in Thai and Chinese traditional medicine for treatment of fever, colds, flu, sore throat, rheumatic diseases, digestive disorders, night blindness, and influenza [29, 30]. Various pharmacological activities of AL as well as its active compounds β-eudesmol, atracylodin, hinesol and atracylon have been demonstrated [26]. With regard to anticancer activity, inhibitory effect of AL on gastric and colon cancer cells were demonstrated [31, 32]. In addition, the potential role of AL and β-eudesmol in CCA has previously been demonstrated both in vitro and in vivo models [33–35]. Both exhibited promising inhibitory activity in CCA-xenografted nude mice with a marked reduction of tumor size and lung metastasis, as well as prolongation of survival time [34]. β-Eudesmol is a member of the class of compounds known as eudesmane, isoeudesmane or cycloeudesmane sesquiterpenoids [36]. Sesquiterpenoids were shown to inhibit phase II detoxification enzymes including superoxide dismutase (SOD), and UDP-glucuronosyltransferase (UGT). The cytotoxic and apoptotic activities on HL-60 cells of sesquiterpenolides (atractylenolide I, AT-I) from the dried rhizome of *Atractylodes ovata* were shown to be through inhibition of Cu-Zn-SOD activity [37]. In addition, the inhibitory effect on UGT activity of the sesquiterpenoid xanthorrhizol, a major component of the essential oil of *Curcuma xanthorrhiza* was demonstrated in an in vivo study [38]. Nevertheless, the cytotoxic activity of sesquiterpenoids through modulation of NQO1 or other detoxifying enzymes has not been fully investigated. We have previously reported that β-eudesmol exerts potent growth inhibitory activity on CCA cells which might be linked to its suppressive effect on heme oxygenase-1 (HO-1) production, STAT1/3 activation, and NF-κB downregulation [39]. Based on this information together with the complex multidirectional biological activity of β-eudesmol, it is also worth investigating its activity on the NQO1 target which is highly expressed in CCA. In the present study, the effect of β-eudesmol on NQO1 suppression-enhanced chemotherapeutic effects of 5-fluorouracil (5-FU) and doxorubicin (DOX) was observed in the human CCA cell line, KKU-100. In addition, the molecular events associated with the inhibition of cell proliferation, cell migration, and induction of apoptosis were investigated.

## Methods

### Chemical and reagents

The cell culture medium Ham’s F12, fetal bovine serum, 0.25% trypsin-EDTA, and penicillin-streptomycin solution were purchased from Gibco BRL Life Technologies (Grand Island, NY, USA). β-Eudesmol and 5-fluorouracil (5-FU) were purchased from Wako Pure Chemical Industries Ltd. (Osaka, Japan). Doxorubicin hydrochloride (DOX) was obtained from Boryung Pharm (Seoul, South Korea). Dimethyl sulfoxide (DMSO) was purchased from Lab-Scan Analytical Science (Dublin, Ireland). β-Eudesmol was dissolved in 100% ethanol. 5-FU and DOX were dissolved in 100% DMSO. Concentrations of both solvents used in all experiments were less than 1% (*v*/v).

All reagents used for NQO1 activity assay and sulforhodamine B (SRB) assay were obtained from Sigma Chemical Co. Ltd. (St. Louis, MO, USA). The primary rabbit polyclonal IgG NQO1 antibody (1:2500) (ab34173) was purchased from Abcam (Cambridge, MA, USA). The rabbit polyclonal IgG Bax (1:2500) (#sc-493), mouse monoclonal IgG Bcl-2 (1:1000) (#sc-7382) and mouse monoclonal IgG β-actin (1:2500) (#sc-1616), and the secondary horseradish peroxidase (HRP)-linked antibodies (goat anti-rabbit IgG; #sc-2004 and goat anti-mouse IgG; #sc-2005 at 1:5000 dilution) were obtained from Santa Cruz Biotechnology, Inc. (California, USA). The Amersham™ ECL™ Prime Western Blotting Detection Reagent was obtained from Amersham Biosciences Crop. (NJ, USA). Annexin V-FITC apoptosis detection kit (Cat no. 420201) and 4′-6-diamidino-2-phenylindole (DAPI) (Cat no. 422801) were purchased from BioLegend Inc. (San Diego, CA, USA). CellEvent™ Caspase-3/7 green detection reagent (Cat no. C10723) was obtained from Invitrogen (Thermo Fisher Scientific Inc., Massachusetts, USA).

### Cell lines and cell culture

The human CCA cell line, KKU-100, with a high expression level of NQO1 was used in the study [23]. The cell was originally derived from intrahepatic CCA tissue of a patient and was kindly provided by Professor Banchob Sripa, Department of Pathology, Faculty of Medicine, Khon Kean University. The cell was routinely cultured in complete medium consisting of Ham’s F12 supplemented with 10% fetal calf serum, 12.5 mM HEPES (pH 7.3), 100 U/mL penicillin G, and 100 μg/mL streptomycin and maintained at 37 °C under an atmosphere of 5% CO_2_. Culture medium was renewed every 3 days. Cells were trypsinized with 0.25% trypsin-EDTA and subcultured in the same culture medium.

### NAD(P)H-quinone oxidoreductase 1 (NQO1) activity assay

The NQO1 activity assay was performed according to the previously described method [40]. In brief, the KKU-100 cells were cultured overnight at 37 °C in a 96-well microtiter plate (7.5 × 10^3^ cells/well). The cells were exposed to various concentrations of β-eudesmol (0, 1, 10, 30, and 100 μM) and dicoumarol (1 μM), a potent pharmacological NQO1 enzyme inhibitor (37 °C, 24 h). Cells were lysed with 50 μL of 0.8% digitonin in 2 nM EDTA with agitation for 10 min. The assay was performed using menadiol and MTT [3-(4, 5-dimethylthiazol-2-yl)-2,5-diphenyltetrazolium bromide] in the substrate coupling reaction and measured as rate-kinetics at the wavelength of 620 nm. To further investigate the direct effect of β-eudesmol on NQO1 activity, β-eudesmol (1–100 μM) was added to KKU-100 cell lysates. Following incubation (37 °C, 15 min), the NQO1 activity of the lysates was determined. Using the extinction coefficient of MTT formazan of 11,300 M^− 1^ cm^− 1^ at 620 nm and correction for the light path of the microplate, activity of NQO1 is expressed as nmol/min/mg protein. The assay was performed as three independent experiments, triplicate each.

### Western blot analysis

Western blot analysis was performed to determine the expression levels of NQO1 and apoptotic proteins following exposure to various concentrations of β-eudesmol with or without 5-FU or DOX. In brief, KKU-100 cells were washed with PBS and lysed with 1× cell lysis buffer (4 °C) containing 1 mmol/L dithiothreitol (DTT), and 0.1 mmol/L phenylmethylsulfonyl fluoride (PMSF) with vigorous shaking. Following centrifugation at 12,000 g for 30 min, the cell supernatant was collected and stored at − 80 °C until use. The sample was mixed with 5× loading dye buffer and heated at 90 °C for 10 min. Proteins were separated by electrophoresis (in 10% SDS-polyacrylamide gel) and transferred to polyvinylidene difluoride (PVDF) membranes (180 mA, 1 h). The PVDF membranes were blocked with 5% (*w*/*v*) skimmed milk powder in PBS and 0.1% Tween-20 (25 °C, 1 h) and incubated overnight at 4 °C with primary antibodies diluted with PBS and 0.1% Tween-20. The antibodies used were as follows: rabbit polyclonal IgG NQO1 (1:2500), rabbit polyclonal IgG Bax (1:2500), mouse monoclonal IgG Bcl-2 (1:1000) and mouse monoclonal IgG β-actin (1:2500). The primary antibody was removed and the blots were extensively washed with PBS/Tween-20 and incubated (25 °C, 2 h) with the secondary antibodies (horseradish peroxidase goat anti-mouse IgG and goat anti-rabbit IgG at 1:5000 dilution in PBS buffer). After removal of the secondary antibody and washing with PBS/Tween-20, the blots were incubated with the ECL substrate solution. Densities of the specific bands of NQO1, Bcl-2, Bax, and β-actin were visualized and captured by Imagequant™ LAS4000.

### Cell cytotoxicity assay

Cytotoxicity testing was performed using the sulphorhodamine B (SRB) assay. Briefly, KKU-100 cells were seeded onto a 96-well microtiter plate (7.5 × 10^3^ cells/well) and incubated overnight at 37 °C. Cells were exposed to various concentrations of the test compounds (200 μL) for specified periods as follows: (i) β-eudesmol alone (0, 1, 10, 30, and 100 μM) for 24 and 48 h; (ii) 30 μM β-eudesmol in combination with 5-FU (0, 3, 10, 30, and 100 μM) for 24 and 48 h; and (iii) 30 μM β-eudesmol in combination with DOX (0, 0.1, 0.01, 1, and 10 μM) for 24 and 48 h. The cells were fixed with 100 μL of ice-cold 10% trichloroacetic acid (TCA) at 4 °C for at least 1 h. TCA was removed and the cells were washed 5 times with distilled water. After 10 min of air drying, 50 μL of 0.4% sulforhodamine in 1% acetic acid was added and the cell suspension was incubated at 25 °C for 30 min. Cells were rinsed 3–4 times with 1% acetic acid and air dried at 25 °C for 1 h. Finally, the adhered cells were dissolved in 10 mM Tris base (200 μL) and the plate was shaken for 20 min. The absorbance of cell suspension was measured at the wavelength of 540 nm. Cell growth inhibition was expressed in term of percentage of untreated control absorbance. The IC_50_ (concentration that inhibits cell growth by 50%) was estimated from concentration-response curve analysis using Prism 5 program (GraphPad Software, San Diego, CA, USA).

### Cell migration by wound healing assay

Cell migration was assessed using wound healing assay according to the previously described method [41]. Briefly, KKU-100 cells (1.5 × 10^5^ cells/well) were seeded onto a 24-well microtiter plate and allowed to grow overnight at 37 °C in Ham’s F12 medium supplemented with 10% fetal calf serum. A scratch wound was made using a sterile 200 μL pipette tip and the scratched cells were washed twice with PBS to remove any detached cells. Cells were exposed to various concentrations of the test compounds (200 μL) for 48 h as follows: (i) β-eudesmol (30 μM) in combination with 5-FU (30 μM); and (ii) β-eudesmol (30 μM) in combination with DOX (0.1 μM). The width of the wound outline was monitored under a phase-contrast microscope. The closing of the scratched wound was determined by capturing the denuded area along the scratch using Image-Pro Plus software (Media Cybernetics, LP, USA).

### Cell apoptosis analysis and caspase 3/7 activation

The potentiating effect of β-eudesmol (30 μM) on 5-FU (30 μM) or DOX (0.1 μM)-induced apoptosis in KKU-100 cells was investigated using Annexin V-FITC Apoptosis Detection Kit. Following a 24 h incubation, cells were washed twice with cold BioLegend’s cell staining buffer and resuspended in Annexin V binding buffer to obtain the cell density of 5 × 10^5^ cells/mL. FITC Annexin V and propidium iodide solution (5 μL each) were added and the cells were incubated at 25 °C for 15 min in the dark. Apoptotic cells were analyzed by flow cytometry (BD FACSCan to II, BD Biosciences, San Joes, CA, USA). Intact nuclei within apoptotic cells were stained and observed by DAPI method according to the manufacturer’s procedure. Following exposure to β-eudesmol in combination with 5-FU or DOX for 24 h, KKU-100 cells (1.5 × 10^5^ cells/well of a 24 well microtiter plate) were washed with PBS, fixed with iced methanol for 10 min, and stained with 1 μg/mL DAPI for 15 min (25 °C in the dark). Results are expressed with complementary nuclear morphological observations gathered using fluorescence microscope (ZOE™ fluorescent Cell imager: BIO-RAD, California, USA).

The effects of β-eudesmol in combination with 5-FU or DOX on stimulation of caspase 3 and caspase 7 of the apoptosis pathway in the KKU-100 cells were investigated using CellEvent™ Caspase 3/7 Green detection assay. Caspase 3/7 Green is a novel fluorogenic substrate that activates caspase 3 and caspase 7 which constitute a hallmark of the apoptotic process. The reagent consists of a four amino acid peptide (DEVD) conjugated to a nucleic acid binding dye. This cell-permeant substrate is intrinsically non-fluorescent because the DEVD peptide inhibits the ability of the dye to bind to DNA. After activation of caspase 3 or caspase 7 in apoptotic cells, the DEVD peptide is cleaved, enabling the dye to bind to DNA and produce a bright, fluorogenic response with an absorption/emission maxima of ~ 502/530 nm. The KKU-100 cells (1.5 × 10^5^ cells) were mixed with the test compounds as described above and seeded onto each well of a 24-well culture plate. Following incubation (37 °C under 5% CO_2_ for 24 h), cells were washed with PBS and fixed with iced methanol (10 min). CellEvent™ caspase 3/7 Green Detection Reagent was added to each well at a final concentration of 10 mM and incubated at 25 °C for 30 min in the dark. Cells were observed under a light and a fluorescence microscope (ZOE™ fluorescent Cell imager: BIO-RAD, California, USA).

### Statistical analysis

Statistical analysis was performed using the Prism 5 program (GraphPad Software, San Diego, CA, USA). Quantitative data are expressed as mean ± SD of three independent assays, triplicate each. Comparison of difference in data of two dependent quantitative groups was performed using paired t-test at a statistical significance level of *p* < 0.05.

## Results

### The sensitivity of NQO1 to β-eudesmol

**Fig. 1:**
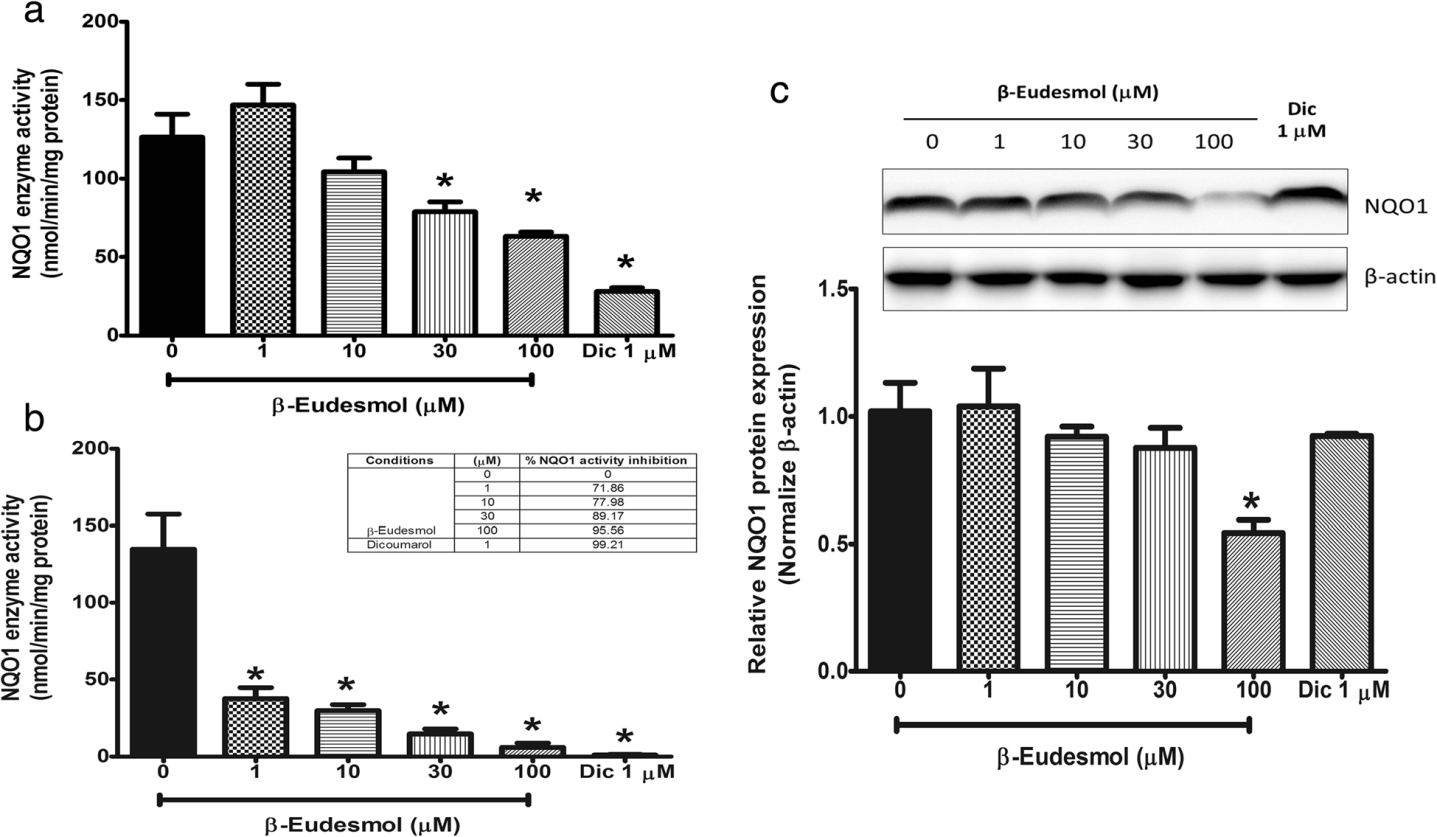
Concentration responses of NQO1 suppression by β-eudesmol KKU-100 cells were seeded onto a 96-well microtiter plate overnight and cells were exposed to β-eudesmol (1–100 μM) and dicoumarol as a potent positive NQO1 inhibitor (1 μM) for 24 h. **a** The effect of β-eudesmol on NQO1 enzyme activity of KKU-100 cells analyzed by the enzymatic method, **b** The effect of β-eudesmol on NQO1 enzyme activity of cell lysates analyzed by the enzymatic method, and **c** The effect of β-eudesmol on the expression of the NQO1 protein by Western blot analysis using β-actin as an internal control for equal protein loading. The relative bar that was normalized with β-actin of each band is shown below. Each bar represents mean ± SD of three independent experiments, triplicate each. * indicates statistically significant difference with untreated control (paired t-test, *p* < 0.05)

β-Eudesmol at the concentration range 1–100 μM significantly inhibited NQO1 enzyme activity of the KKU-100 cells in a concentration-dependent manner (Fig. 1a). Dicoumarol was the most potent inhibitor of NQO1 activity (77.96% inhibitory effect at 1 μM). For β-eudesmol, significant inhibitory activity on NQO1 activity was observed at 30 and 100 μM (37.68 and 50.06%, respectively). β-Eudesmol also produced an inhibitory effect on the NQO1 activity of the cell lysates in a concentration-dependent manner (Fig. 1b). The inhibitory effect occurred within 15 min of exposure. At the lowest concentration (1 μM), the enzyme activity was inhibited by 71.86% and almost complete inhibitory effect (95.56%) occurred at the highest concentration (100 μM). Furthermore, Western blot analysis was performed to verify whether the suppressive action of β-eudesmol towards NQO1 activity was due to direct inhibition of the enzyme activity or modulation of its expression. These results showed that the NQO1 protein expression was significantly suppressed only when the cells were exposed to the highest concentration (100 μM) of β-eudesmol (Fig. 1c).

### Potentiating effect of β-eudesmol on cytotoxic activities of 5-FU and DOX

**Fig. 2:**
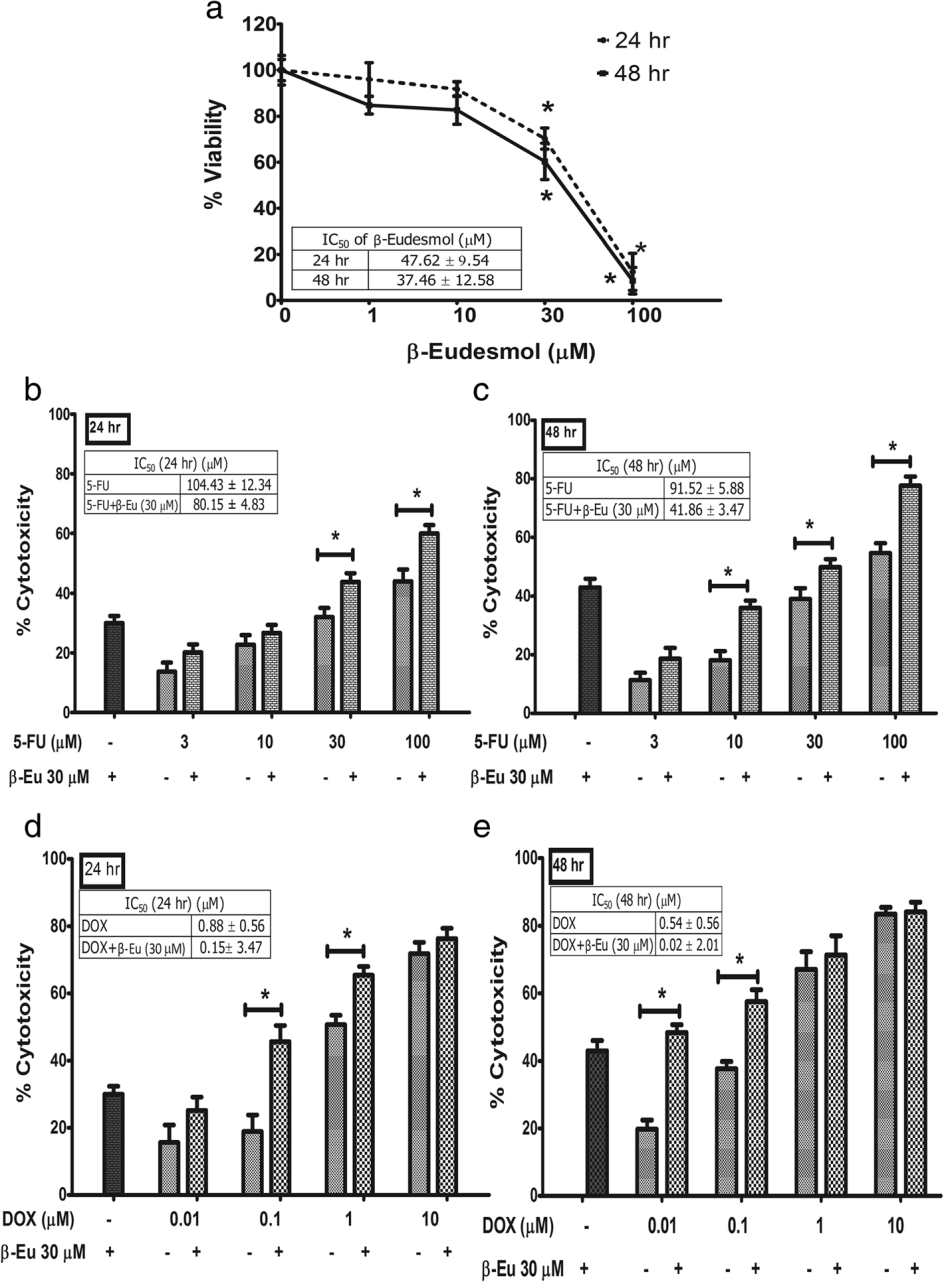
Potentiating effect of β-eudesmol on cytotoxic activities of 5-FU and DOX. KKU-100 cells were seeded onto a 96-well microtiter plate overnight and cells were treated with β-eudesmol (1, 10, 30 and 100 μM) in the absence and presence of 5-FU or DOX for 24 and 48 h. Cell death was determined using SRB assay. **a** KKU-100 cells were incubated with β-eudesmol (1, 10, 30 and 100 μM) for 24 and 48 h. The results are presented as the percentage of viable cells. **b**-**e** The cytotoxic effect of β-eudesmol in combination with chemotherapeutic agents was determined. **b**-**c** KKU-100 cells were treated with β-eudesmol (30 μM) in the absence and presence of 5-FU (3, 10, 30 and 100 μM) for 24 and 48 h. **d**-**e** KKU-100 cells were treated with β-eudesmol (30 μM) in the absence and presence of DOX (0.01, 0.1, 1 and 10 μM) for 24 and 48 h. Each bar represents mean ± SD of three independent experiments, triplicate each. * indicates statistically significant difference with each drug alone (t-test, *p* < 0.05). β-Eu; β-Eudesmol, 5-FU; 5-Fluorouracil, DOX; Doxorubicin

To investigate whether the inhibitory activity of β-eudesmol on NQO1 activity and protein expression in the CCA cells resulted in an enhanced sensitivity of the cells to chemotherapeutic agents, the KKU-100 cells were incubated with β-eudesmol in the absence and presence of 5-FU or DOX for 24 or 48 h. β-Eudesmol potently inhibited cell growth with mean (±SD) IC_50_ of 47.62 ± 9.54 and 37.46 ± 12.58 μM following 24 and 48 h exposure (Fig. 2a). The concentration of β-eudesmol below the IC_50_ (30 μM) was therefore used in subsequent experiments (potentiating effect on cytotoxic activities, inhibitory effect on cells migration, and enhancing the effect on cell apoptosis) to avoid the complete cytotoxic effect of β-eudesmol. Combination of β-eudesmol with 5-FU or DOX resulted in a markedly enhanced cytotoxic effect of each drug in concentration- and time-dependent manners (Fig. 2b-e). The cytotoxic activity of 5-FU was increased by 24 and 55% at 24 and 48 h of β-eudesmol exposure, respectively. The enhancement effect of β-eudesmol on the cytotoxic activity of DOX by greater than 80% was observed following 24 and 48 h of exposure.

### Inhibitory effects of β-eudesmol in combination with 5-FU or DOX on CCA cell migration

**Fig. 3:**
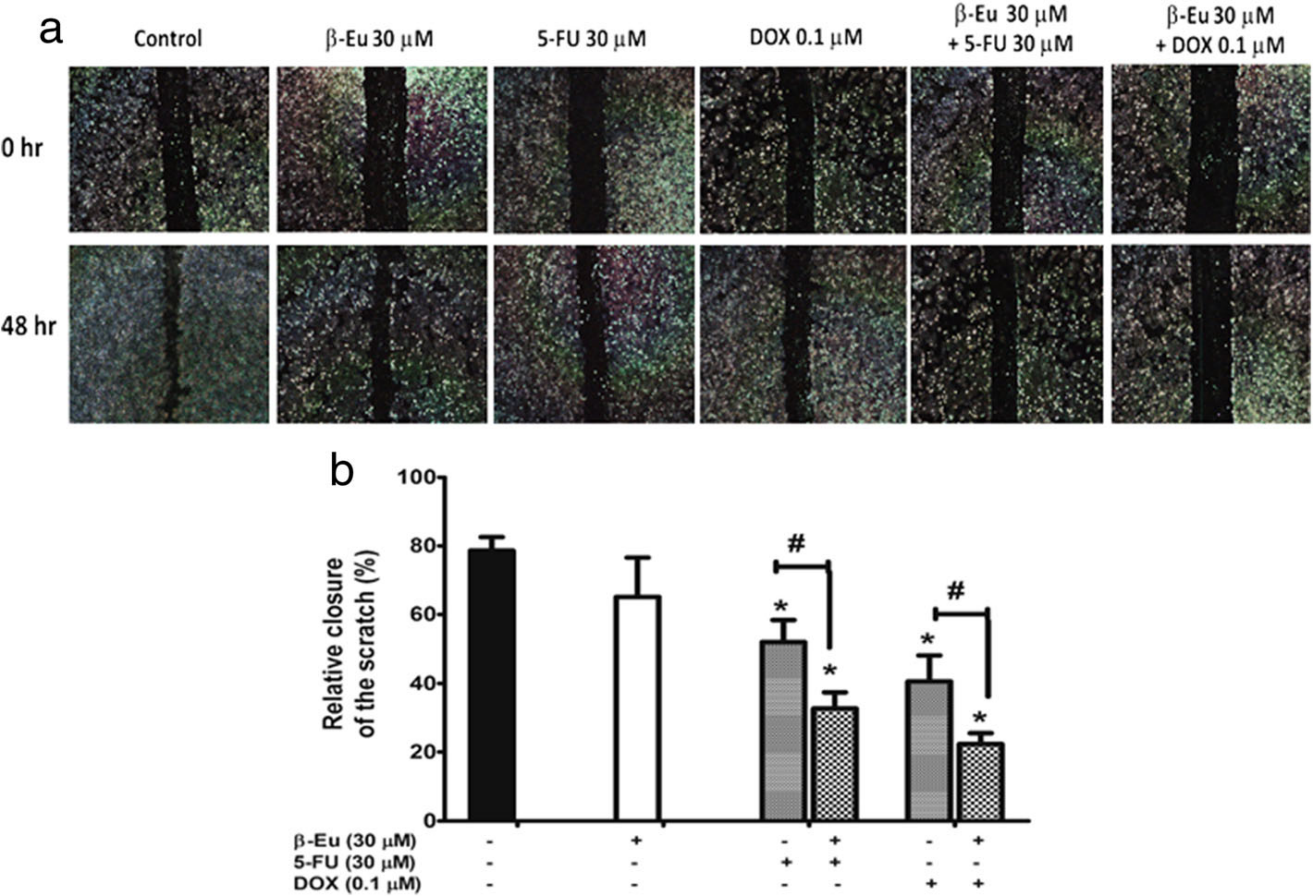
Inhibitory effects of β-eudesmol in combination with 5-FU or DOX on CCA cells migration*.* Scratched wounds of monolayer KKU-100 cells were exposed to β-eudesmol (30 μM), 5-FU (30 μM), DOX (0.1 μM), and the combination of β-eudesmol (30 μM) with 5-FU (30 μM) or DOX (0.1 μM). Cell migration was monitored under phase-contrast microscopy (× 4 magnification). Representative images of wound healing were obtained at the time of the scratch and 48 h later (**a**). **b** The graph shows the level of cell migration into the wound scratch quantified as the percentage of wound closure at 48 h. Each bar represents mean ± SD of three independent experiments, triplicate each. * indicates statistically significant difference with each drug alone (paired t-test, *p* < 0.05). *β-Eu = β-Eudesmol, 5-FU = 5-Fluorouracil, DOX = Doxorubicin*

Based on the wound closure assay, closure of the scratched wound of the monolayer culture of KKU-100 cells significantly occurred following exposure to β-eudesmol (30 μM) with or without 5-FU (30 μM) or DOX (0.1 μM) for 48 h (Fig. 3a and b).

### Enhancing effect of β-eudesmol on apoptotic activities of 5-FU and DOX on CCA cells

**Fig. 4:**
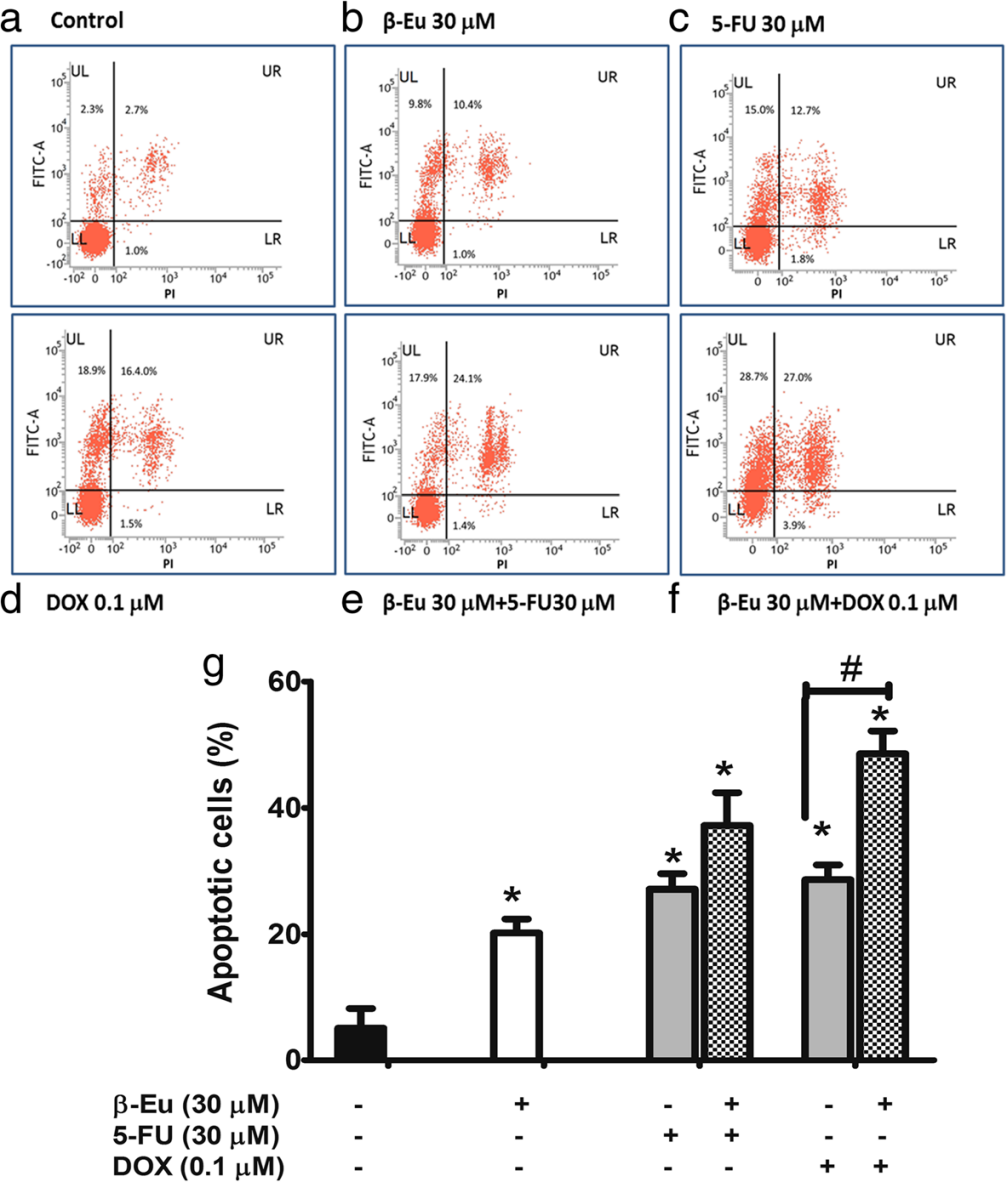
Enhancing the effect of β-eudesmol on apoptotic activities of 5-FU and DOX on CCA cells. Flow cytometry analyses of KKU-100 cells using double staining with annexin V (annexin V, vertical line) and propidium iodide (PI, horizontal line). **a** control, **b** β-eudesmol (30 μM), **c** 5-FU (30 μM), **d** DOX (0.1 μM), **e** combination of β-eudesmol (30 μM) with 5-FU (30 μM), and **f** combination of β-eudesmol (30 μM) with DOX (0.1 μM) for 24 h. Flow cytometry apoptotic results are shown in four subpopulations which indicate: early apoptotic cells (upper left), late apoptotic cells (upper right), normal cells (lower left) and necrotic cells (lower right). **g** The levels of KKU-100 cell apoptosis expressed as the percentage of apoptotic cells are depicted in the graph (mean ± SD averaged from three independent experiments, triplicate each). *p < 0.05 vs untreated control. ^#^*p* < 0.05 vs chemotherapeutic agents alone. * indicates statistically significant difference with each drug alone (paired t-test, *p* < 0.05). *β-Eu = β-Eudesmol, 5-FU = 5-Fluorouracil, DOX = Doxorubicin*

**Fig. 5:**
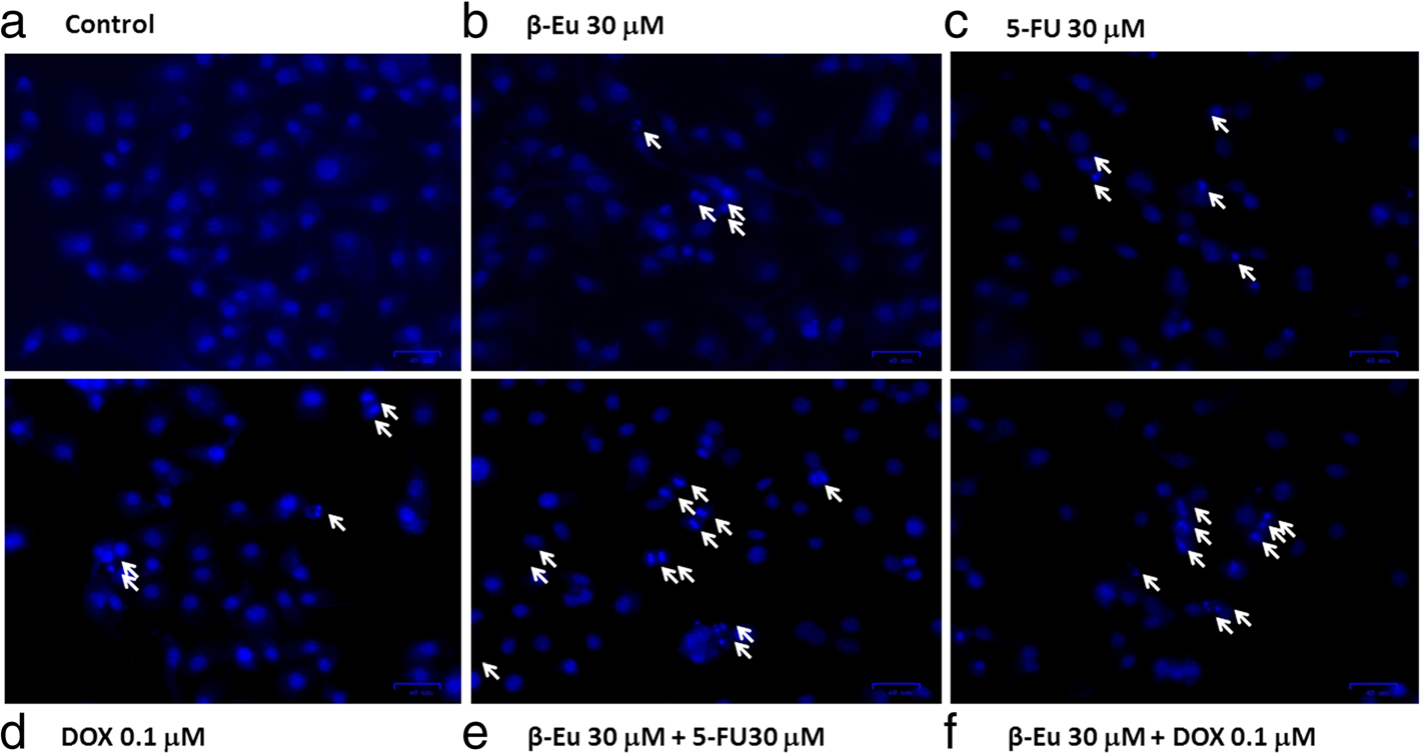
Apoptotic bodies of KKU-100 CCA cells following treatment with β-eudesmol, 5-FU, and DOX, and the combination of β-eudesmol with 5-FU or DOX by DAPI staining. **a** control, **b** β-eudesmol (30 μM), **c** 5-FU (30 μM), **d** DOX (0.1 μM), **e** combination of β-eudesmol (30 μM) with 5-FU (30 μM), and **f** combination of β-eudesmol (30 μM) with DOX (0.1 μM) for 24 h. Apoptotic bodies were observed under inverted fluorescence microscopy (Scale bars represent 40 μm). *β-Eu= β-Eudesmol, 5-FU= 5-Fluorouracil, DOX= Doxorubicin*

**Fig. 6:**
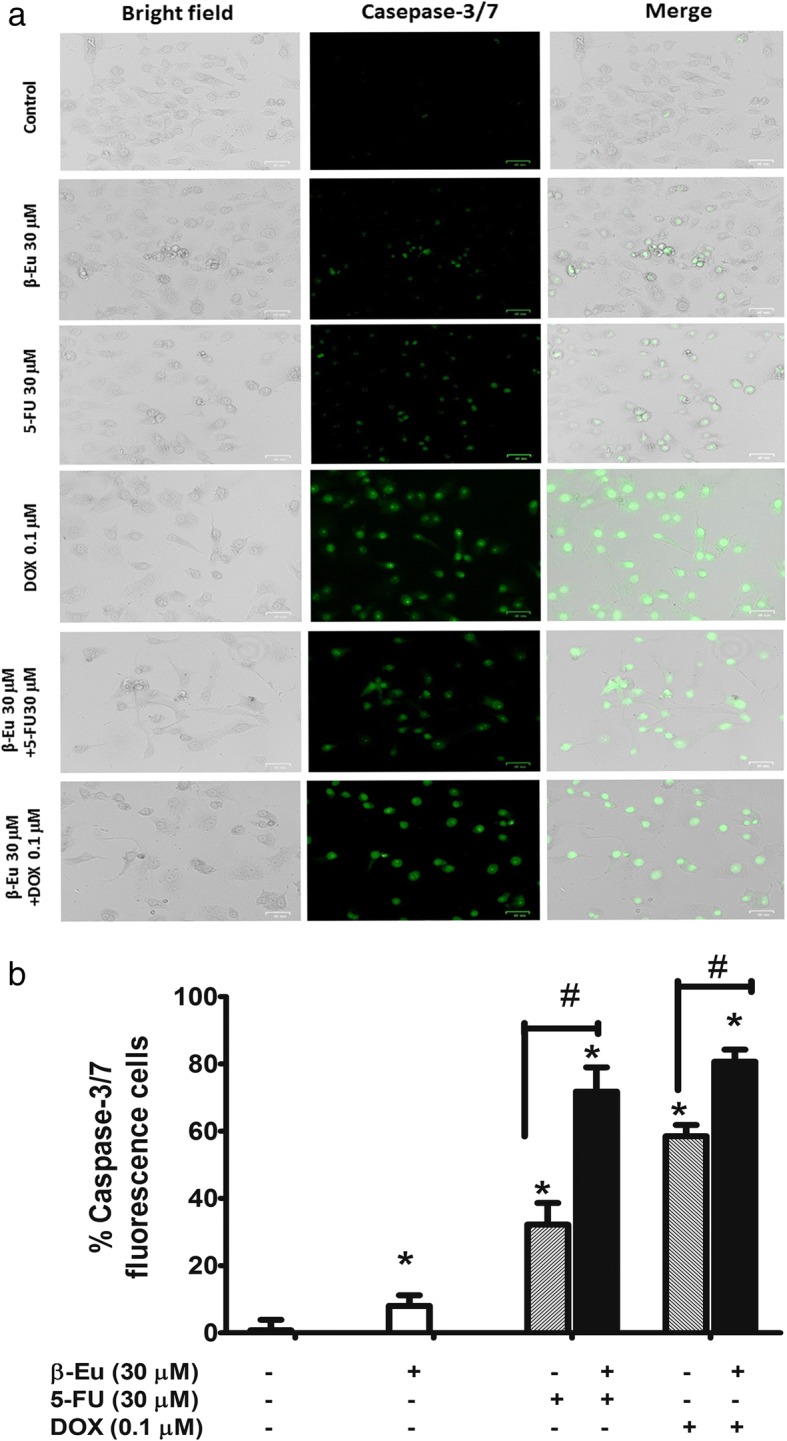
Caspase-3/7 activation in KKU-100 cells following treatment with β-eudesmol, 5-FU, and DOX, and the combination of β-eudesmol and 5-FU or DOX detected by fluorescence microscopy. **a** KKU-100 cells were treated with 30 μM of β-eudesmol, 30 μM of 5-FU, 0.1 μM of DOX and β-eudesmol (30 μM) combined with 5-FU (30 μM) or DOX (0.1 μM) for 24 h and labeled with CellEvent® Caspase 3/7 Green Detection Reagent and examined under the fluorescence microscope. Left panel, the grey image reveals KKU-100 CCA cells in the field of view. Middle and right panel show apoptotic caspase 3/7 positive cells green fluorescent in the same area of interest. Data are representative of at least 4–5 randomly selected fields’ images with similar results. Morphological changes were evaluated under a microscope. There were shrink and blabbing cells treated with β-eudesmol with or without 5-FU or DOX, indicating apoptosis induction. The scale bar corresponds to 40 μm. **b** The graph shows the level of caspase-3/7 activity as the percentage of caspase-3 /7 fluorescent cells expression at 24 h. Data are presented as mean ± SD of three independent experiments, triplicate each. * indicates statistically significant difference with each drug alone (paired t-test, *p* < 0.05). *β-Eu= β-Eudesmol, 5-FU= 5-Fluorouracil, DOX= Doxorubicin*

Annexin V/PI and DAPI staining assay was used to further examine whether KKU-100 cell death following exposure to β-eudesmol in combination with 5-FU or DOX was associated with cell apoptosis (Fig. 4, 5 and 6). Percentage of annexin V/PI stained cells following exposure to β-eudesmol in combination with 5-FU or DOX (Fig. 4e, f) was compared with that following exposure to each compound alone (Fig. 4b-d). The calculated percentage of the stained cells is shown in Fig. 4g. The inducing effect of β-eudesmol (30 μM) alone on cell apoptosis was relatively weak but was markedly increased in the presence of 5-FU or DOX. This observation of the enhancing cytotoxic activities of 5-FU or DOX by β-eudesmol through induction of cell apoptosis was supported by the analysis of cell morphology after staining with DAPI (Fig. 5). The number of apoptotic cells, nuclear condensation, and fragmentation were more prominent following exposure to the combination of β-eudesmol and 5-FU or DOX (Fig. 5e, f) compared with 5-FU or DOX alone (Fig. 5b-d).

### Enhancing effect of β-eudesmol on caspase activation on CCA cells by 5-FU and DOX

To demonstrate the mechanisms underlying apoptotic induction, activation of caspase 3/7 of the cell apoptosis cascade was investigated following exposing the KKU-100 cells to β-eudesmol in combination with 5-FU or DOX. The caspase 3/7 activity of the cells in the presence of β-eudesmol alone (30 μM) was slightly but significantly increased compared with untreated control cells. However, the activity was markedly increased following exposure to β-eudesmol (30 μM) in combination with 5-FU (30 μM) or DOX (0.1 μM) for 24 h (Fig. 6). These results suggested that combination of β-eudesmol and 5-FU or DOX enhanced KKU-100 cell apoptosis through caspase 3/7 activation.

### Potentiating effect of β-eudesmol on suppression Bcl-2 and Bax protein expression of CCA cells by 5-FU and DOX and the involvement of mitochondrial pathway

**Fig. 7:**
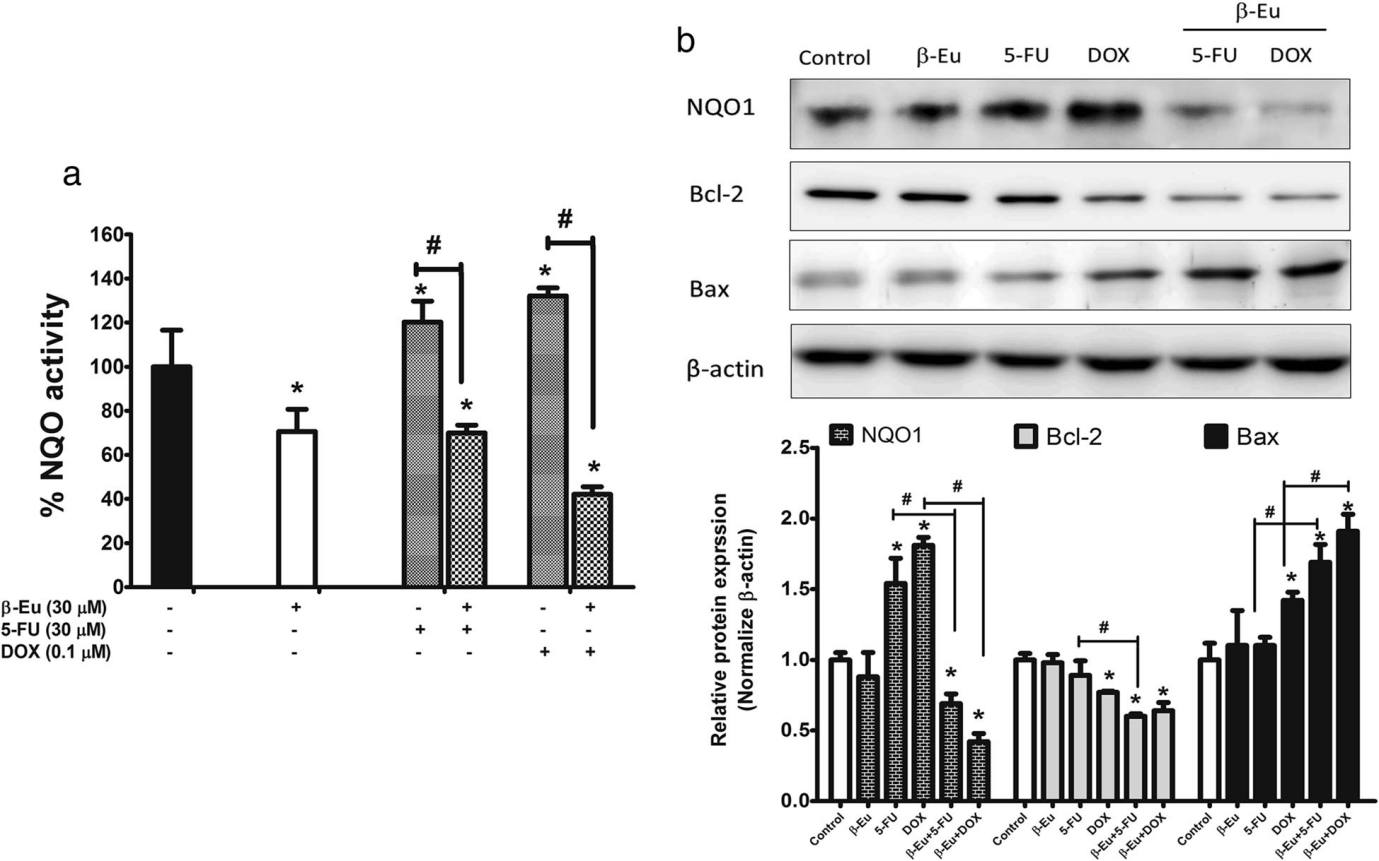
Potentiating effect of β-eudesmol on suppression Bcl-2 and Bax protein expression by 5-FU and DOX on CCA cells. **a**-**b** KKU-100 cells were exposed to β-eudesmol (30 μM), 5-FU (30 μM), DOX (0.1 μM), and combination of β-eudesmol (30 μM) with 5-FU (30 μM) or DOX (0.1 μM) for 24 h. **a** Effect of β-eudesmol on potentiation of 5-FU and DOX sensitivity on NQO1 enzyme activity analyzed by enzymatic method. **b** Equal amounts of total protein were examined by Western blot analysis, with appropriate antibodies. β-actin was used as a loading control. Relative band intensities of NQO1, Bcl-2, and Bax were shown. Data are presented as mean ± SD of three independent experiments, triplicate each. * indicates statistically significant difference with each drug alone (paired t-test, *p* < 0.05). *β-Eu= β-Eudesmol, 5-FU= 5-Fluorouracil, DOX= Doxorubicin*

The NQO1 enzyme activity and protein expression in KKU-100 cells were determined to examine whether the potentiating effect of β-eudesmol on cytotoxic activities of 5-FU or DOX was mediated through suppression of oxidative stress produced by NQO1. Cells were treated with β-eudesmol with or without 5-FU (30 μM) or DOX (0.1 μM) for 24 h. 5-FU and DOX alone significantly enhanced NQO1 activity and protein expression in KKU-100 cells. In the presence of β-eudesmol however, enzyme activity and protein expression were markedly decreased (Fig. 7a and b).

In order to further confirm the involvement of the mitochondrial pathway in β-eudesmol enhanced chemosensitivity of KKU-100 cells through the induction of cell apoptosis, the expression levels of Bcl-2 family proteins Bcl-2 and Bax were investigated by Western blot analysis. β-Eudesmol (30 μM) alone did not alter Bax and Bcl-2 protein expression. On the other hand, the combination of β-eudesmol with 5-FU (30 μM) or DOX (0.1 μM) significantly increased the Bax/Bcl-2 expression ratios (1.23 vs. 2.81 and 1.84 vs. 2.98 for 5-FU alone vs. 5-FU + β-eudesmol and DOX alone vs. DOX + β-eudesmol, respectively) (Fig. 7b). These results suggested that β-eudesmol-potentiated cytotoxic activity of 5-FU and DOX was mediated through induction of apoptosis by Bcl-2 protein family in the mitochondrial pathway.

## Discussion

Since the up-regulation of NQO1 in certain types of solid tumor including CCA was associated with poor prognosis [14–19], compounds targeting NQO1 would be of therapeutic potential. Previous studies have suggested possible role of NQO1 suppression-enhanced chemosensitivity of the cancer cells. Dicoumarol, a potent inhibitor of the NQO1 enzyme, has been shown to increase sensitivity of CCA cells to chemotherapeutic agents [22]. Furthermore, NQO1 silencing by gene knockdown in conjunction with chemotherapeutic agents has been shown to suppress the replication capacity of CCA cells [23]. In the present study, β-eudesmol suppressed NQO1 enzyme activity in KKU-100 cells with moderate potency compared with dicoumarol, the most potent inhibitor of NQO1 [28]. Direct addition of β-eudesmol to cell lysates significantly inhibited NQO1 activity at all concentrations. At highest concentration (100 μM, 95.56% inhibition), the extent of the inhibitory activity was almost similar to that of dicoumarol (1 μM, 99.21% inhibition). On the other hand, significant suppression of NQO1 protein expression was observed only at the highest concentration (100 μM) compared with that inhibited enzyme activity both in the cells (at the lowest concentration of 30 μM) and cell lysates (at the lowest concentration of 1 μM). Dicoumarol has been shown to exert no or little effect on CCA NQO1 protein expression [42]. It is possible that β-eudesmol-mediated NQO1 suppression was through direct inhibition of the enzyme activity with similar mechanism of that observed with dicoumarol. The suppression of NQO1 activity by dicoumarol is a consequence of its competition with NAD(P)H for binding to NQO1 and prevention of electron transfer to FAD co-factor, allowing the quinone substrate to bind the enzyme and to be reduced [43]. It is well established that NQO1 functions as a detoxification and antioxidant enzyme that protects cells from oxidative stress. Additionally, it also functions as a p53 wild-type stabilizer by interference with 20s proteasome-mediated degradation of p53 [44]. P53 is a tumor suppressor gene which functions in response to stimulation by DNA damage, oxidative stress, or cell cycle abnormalities [44, 45]. The anticancer and apoptotic activity of P53 involves several mechanisms [46]. In the present study, β-eudesmol was shown to enhance chemosensitivity-induced apoptosis of CCA cells which was linked with suppression of NQO1 activity. So far, the previously reported role of NQO1 suppression on p53 modulation and cell apoptosis remains controversial. Inhibition of NQO1 by natural inhibitor curcumin has been shown to suppress p53 protein levels and p53-induced apoptosis of cancer cells in the NQO1-dependent pathway [26]. On the other hand, dicoumarol or NQO1 knockdown cells was shown to enhance p53 protein levels which was associated with induction of apoptosis in CCA [22, 23] and urogenital cancer cells [20]. The CCA-KKU-100 cell used in the present study was shown to express both the wild-type full-length p53 and the splicing variant of the truncated p53 protein [47]. Interestingly, our results showed that the potentiating effect of NQO1 suppression by β-eudesmol on the cytotoxicity and apoptotic activity of 5-FU and DOX occurred even in such the CCA cells with a high expression ratio of mutant p53/wild-type p53. The exact molecular action on NQO1 activity of β-eudesmol needs further investigation. It is yet to investigate the chemosensitizing effect of NQO1 suppression by β-eudesmol on CCA cells which express other p53 mutation variants.

5-FU and DOX are widely used for chemotherapy of several types of cancer including CCA. However, the effectiveness of both drugs in the treatment of recurrent/metastatic cancers is limited due to acquired or intrinsic resistance of the cancer cells. β-Eudesmol was shown to suppress NQO1 enzyme activity and protein expression and thereby, potentiating the cytotoxic activity of both drugs in KKU-100 cells. Combination of both conventional drugs with β-eudesmol may be an effective therapeutic strategy for targeting chemoresistant CCA as well as improving therapeutic efficacy and minimizing toxicity of conventional drugs. The enhancement of CCA cell sensitivity has also been reported when conventional chemotherapeutics are used in conjunction with NQO1 knockdown [23].

Metastasis is one of the most important characteristics of cancers indicating poor prognosis and death in cancer patients. The process reflects the ability of cancer cells to break away from the main tumor and enter the bloodstream or lymphatic system. The key steps include degradation of tumor extracellular matrix, cell invasion, and cell migration [48]. Inhibitors of these metastasis-associated processes would, therefore, provide a significant impact on cancer chemoprevention and chemotherapy. Previous studies have shown that NQO1 is one of the enzymes that play an important role in cancer cell migration and invasion of human aortic vascular smooth muscle cells. The NQO1 inhibitor dicoumarol or NQO1 knockdown was shown to suppress matrix metallopeptidase 9 (MMP 9) expression and tumor necrosis factor α (TNF-α)-induced cell migration [49]. In the present study, treatment of KKU-100 cells with β-eudesmol markedly decreased cancer cell migration mediated by 5-FU or DOX. It is of note that this antimigratory effect of β-eudesmol was detected at a concentration that significantly inhibited NQO1 expression (> 40%) with minimal cytotoxic effect to KKU-100 cells. The chemosensitivity enhancing effect of β-eudesmol on the CCA cell could at least in part, be a consequence of its inhibitory effect on CCA cell metastasis.

The process of programmed cell death or apoptosis is generally characterized by a programmed sequence of events leading to the eradication of cells without releasing damaging substances into the surrounding area [50]. Initiation of apoptosis process therefore, benefits cancer cells. The mechanism through which β-eudesmol mediated suppression of NQO1-induced cell death was further explored in the present study. β-Eudesmol was shown to significantly enhance proapoptotic activities of both 5-FU and DOX. During the steps of apoptosis, the cells shrunk despite undamaged membranes. Cells with DNA fragmentation, condensed chromatin, and nuclear pyknosis were detected by DAPI. Combination of β-eudesmol and 5-FU or DOX markedly increased apoptotic bodies. Induction of cell apoptosis by β-eudesmol was also observed in other cancer cells, i.e.*,* human hepatocellular carcinoma [51] and human leukemia cells [52]. Apoptosis process is finely regulated at gene level resulting in the orderly and efficient removal of damaged cells such as those occurring following DNA damage or during development. The balance between the proapoptotic (Bax) and anti-apoptotic (Bcl-2) protein regulators is a critical key point to determine cell apoptosis. The apoptotic activity of various compounds has been evaluated by measuring caspase activity particularly caspase 3/7 activity which is the final step in both the intrinsic or extrinsic pathways of apoptosis [53]. In this study, the combination of β-eudesmol and 5-FU or DOX significantly activated caspase 3/7 activity compared to each drug alone. The β-eudesmol-induced enhacement of chemosensitivity of CCA cells by promoting their apoptosis was shown to be associated with increase of the Bax/Bcl-2 ratio and caspase activation. The Bcl-2 family is a key factor in the regulation of cell homeostasis which is directly associated with cell survival and cell death. The process that promotes pro-apoptotic factor Bax expression or/and decreases anti-apoptotic factor Bcl-2 expression results in the release of cytochrome to the cytosol and subsequently, initiation of caspase 9 and caspase 3 cascades, leading to cell apoptosis [39, 40]. .Our results indicated that β-eudesmol in combination with 5-FU or DOX stimulated the expression of Bax protein while decreasing the expression of Bcl-2.

## Conclusions

The cytotoxic activity of β-eudesmol was at least in part, mediated through suppression of NQO1. Induction of cell apoptosis and activation of caspase 3/7, up-regulation of Bax and down-regulation of Bcl-2 proteins could contribute to its cytotoxic and proapoptotic activity. β-Eudesmol may serve as a potential anti-CCA candidate particularly when used in combination with conventional chemotherapeutics. Further investigations in animal models are needed to confirm its potential clinical use.

## Abbreviations

5-FU: 5-Fluorouracil
CCA: Cholangiocarcinoma
DMSO: Dimethyl sulfoxide
DOX: Doxorubicin
DTT: Dithiothreitol
FBS: Fetal bovine serum
NQO1: NAD(P)H-quinone oxidoreductase1
PMSF: Phenylmethylsulfonyl fluoride
PVDF: Polyvinylidene difluoride
β-Eu: β-Eudesmol
Tween-20: Polyethylene glycol sorbitan monolaurate
